# miRNA-503 inhibition exerts anticancer effects and reduces tumor growth in mesothelioma

**DOI:** 10.1186/s13046-025-03283-0

**Published:** 2025-02-22

**Authors:** Miriam Piccioni, Francesco Di Meo, Anna Valentino, Virginia Campani, Maddalena Arigoni, Mirella Tanori, Mariateresa Mancuso, Rossana Cuciniello, Marco Tomasetti, Federica Monaco, Gaia Goteri, Enrico P. Spugnini, Raffaele A. Calogero, Giuseppe De Rosa, Gianfranco Peluso, Alfonso Baldi, Stefania Crispi

**Affiliations:** 1 Institute of Biosciences and Bio-Resources, CNR, Naples, Italy; 2 Research Institute on Terrestrial Ecosystems, CNR, Naples, Italy; 3 Department of Life Health Sciences and Health Professions, Link Campus University, Rome, Italy; 4 Department of Molecular Biotechnology and Health Sciences, University of Turin, Turin, Italy; 5 Division of Biotechnologies, ENEA, Casaccia Research Center, Rome, Italy; 6 Department of Clinical and Molecular Sciences, Polytechnic University of Marche, Ancona, Italy; 7 Department of Biomedical Sciences and Public Health, Polytechnic University of Marche, Ancona, Italy; 8 Biopulse Srl, Rome, Italy; 9 Department of Pharmacy, University of Naples Federico II, Naples, Italy; 10 Unicamillus, International University of Health and Medical Sciences, Rome, Italy; 11 Present Address: Aix Marseille University, CNRS, INSERM, CIML, Centre d’Immunologie de Marseille-Luminy, Turing Center for Living Systems, Marseille, France; 12 Present Address: Aix Marseille University, CNRS, IBDM, Turing Centre for Living Systems, NeuroMarseille, Marseille, France

**Keywords:** Mesothelioma, miRNA-503 inhibition, Apoptosis, LNPs, Tumor growth inhibition

## Abstract

**Background:**

Malignant mesothelioma (MM) is a rare and aggressive form of cancer that affects the mesothelial surfaces, associated with exposure to asbestos fibres. To date, no cure is available for MM and therapeutically approved treatments are based on the use of platinum compounds often used in combination with other drugs. We have previously analysed the efficacy of a cisplatin/piroxicam (CDDP/P) combined treatment showing that this treatment was able to reduce in vivo tumor growth. Several studies reported that platinum-drug sensitivity in cancer is connected to modulation of the expression of non-coding RNAs. In this study we analysed if the CDDP/P treatment was able to modulate miRNAs expression in MM.

**Methods:**

miRNA sequencing performed on MSTO-211 H cells treated with CDDP with CDDP/P led us to identify miRNA-503 - downregulated by CDDP/P - as a novel miRNA that acts as an oncomiR in MM. The effect of miRNA-503 inhibition was evaluated in vitro in mesothelioma cells analysing apoptosis induction and reduction of cancer properties. Inhibition of miR-503 expression in vivo, was analysed in ectopic mouse model of MM by using LNP encapsulating anti-mir-503 and miR-503 expression was evaluated in human MM samples.

**Results:**

In vitro and in vivo analysis confirmed miR-503 acts as oncogene in MM since its inhibition was able to reduce cell cancer properties and tumor growth in ectopic mouse model of MM. Its expression was found upregulated in human MM patients compared to normal pleura. Bioinformatic analysis indicated BTG1, CCNG1, EDG1, and TIMP2 as putative target genes of miRNA-503. These genes showed an opposite expression compared to miR-503 levels both in cells and in MM samples. Finally, microarray analysis indicated that miR-503 inhibition affected the expression of the well-known MM biomarkers: CXCL8, SERPINE1 and Osteopontin.

**Conclusions:**

Our study is the first reporting an oncomiR role for miR-503 in MM and suggests that its inactivation could have a clinical value in MM patients. This study reveals that miRNA-503 acts as an oncomiR in MM suggesting that its inhibition, through LNP delivery, has the potential to be considered as a novel therapeutic strategy in MM.

**Supplementary Information:**

The online version contains supplementary material available at 10.1186/s13046-025-03283-0.

## Background

MM is a rare and aggressive form of cancer that affects the mesothelium surface of the pleural and peritoneal cavity, and it is primarily associated with exposure to asbestos fibres. Despite the rarity of this disease, MM incidence is increasing worldwide, and it is expected to reach the peak in the next years [1], especially in developing countries where insufficient safety procedures are contemplated for asbestos handling [2].

The prognosis of MM is very poor due to the long latency development (30–40 years), to the diagnosis at a very late stage and to its high chemo-resistance [3]. Currently, a multimodal treatment regimen of chemotherapy, surgery, and radiotherapy provides the best long-term results. For patients affected by unresectable MM, the standard therapy is based on systemic treatment with platinum drugs such as cisplatin (CDDP) or carboplatin, often combined with other drugs such as the antimetabolites pemetrexed and gemcitabine. However, none of these treatments extend patient survival over 1-year. In addition, these treatments often develop drug resistance and determine severe side effects, two aspects that represent the main limit for a successful therapy.

The expected increasing incidence and the poor prognosis of MM, claim for additional studies on the molecular pathogenesis of MM with the aim to develop novel therapeutic strategies. In this context, more promising seems the use of microRNAs (miRNAs) as novel treatment.

miRNAs are small non-coding RNAs of approximately 22 nucleotides, that play a key role in gene expression regulation via RNA interference. miRNAs deregulation acts at transcriptional and translational level and can affect different diseases including cancer [4]. In addition, specific miRNAs, involved in physiological processes, can also have a role in tumor progression. For example, miR-21 is known to regulate adipocyte differentiation, but several studies reported that it has an oncogenic role in many types of cancer including MM [5–7].

In cancer, miRNAs can act as tumor suppressor or as oncogene and in vivo studies demonstrated that ectopic expression or inactivation of specific miRNAs can affect tumor relapse [8].

Deregulation of specific miRNAs has been found also in MM and some miRNAs are tested in clinical trials. For example, miR-16, downregulated in MM, can influence oncogenic properties both in vitro and in vivo when it is re-expressed determining inhibition of cell growth and sensitizing them to drug treatment [9]. Furthermore, this miRNA, delivered in a minicells system (TargomiRs) has been used in the first clinical phase 1 study in MM (ClinicalTtrials.gov: NCT02369198) achieving therapeutic benefit by the patients recruited in the trial [10].

miRNA deregulation has been reported to be involved also in the sensitivity or resistance of cancer cells to cisplatin response and it is known that cisplatin can modulate miRNAs expression [11]. Depending on the cellular context, cisplatin-deregulated miRNAs are able to act on key genes of different pathways including apoptosis, autophagy and hypoxia. In fact, different miRNAs involved in cisplatin response can have opposite effects in different tumors. For example, miR-31 plays a dual role in MM, increasing cisplatin resistance in parental cells and cisplatin sensitivity in miR-31 null cells [12]. On the contrary, upregulation of miR-31 induced cisplatin resistance in ovarian cancer and in Non-Small Lung Cancer, while its inactivation in prostate cancer determines cisplatin resistance [13, 14]. Differently, miR-18a acts as oncomiR in several cancers including MM, in which it is considered a predictive marker for CDDP treatment since its inactivation increases cisplatin sensitivity [15, 16].

The expression of specific miRNAs can be differently modulated by combined treatment based on the use of cisplatin with other drugs. For example, CDDP/pemetrexed treatment in MM patients downregulates the expression of four miRNAs: miR-126, miR-143 and miR-652 and miR-145, while in bladder cancer a different drug combination (CDDP/ paclitaxel) upregulated miR-145 [17, 18].

In this regard the present study aimed to verify if the efficacy of CDDP/P (piroxicam) treatment, a combined treatment previously validated in MM by our group [19, 20], was related to the deregulation of specific miRNAs. To this aim we performed a miRNome analysis that led us to identify miR-503-5p (miR-503), as the putative molecular target of the treatment. Downregulation of miR-503 in vitro determined apoptotic increase and reduction of cancer properties and in vivo its inhibition affected tumor growth. Additionally, miR-503 showed opposite expression in MM patients, thus suggesting its oncogenic role in this cancer. These findings indicate that miR-503, often described as tumor suppressor in other tumors, has a different role in MM. Our study is the first reporting a preclinical evaluation on the efficacy of the miR-503 inhibition in MM.

## Materials and methods

### Chemicals and reagents

Piroxicam (Pfizer, New York, NY) was a 60-mmol/L injectable solution; cisplatin (Pharmacia-Italia, MI, Italy) was a 50 mmol/L injectable solution.

Trypan Blue solution, Thiazolyl Blue Tetrazolium Bromide (MTT) and Crystal violet were purchased by Sigma-Aldrich (St. Louis, MI, USA); Annexin V-FITC Kit was purchased by Miltenyi Biotec B.V. & Co. KG, Bergisch Gladbach, Germany). Culture media and supplements were obtained from Euroclone (Euroclone SpA, Pero, Milan, Italy).

anti-miR-503 (hsa-miR-503-5p inhibitor, assay ID MH10378), nc-miR-503 (miRNA inhibitor Negative Control) and the transfection reagent Lipofectamine RNAiMAX were purchased by Thermo Fisher (Waltham, Massachusetts, USA).

For Lipid NanoParticles (LNPs) formulation, ethanol and other solvents were obtained by Exacta Optech (Italy). 1,2-dioleyl-3-dimethylammonium propane (DODAP) and N-palmitoyl-sphingosine-1-succinyl[methoxy(polyethylene glycol)2000] (PEG2000-Cer16) were obtained by Avanti Polar Lipids. Disteroylphosphatidylcholine (DSPC) was kindly offered from Lipoid GmbH (Cam, Switzerland). Cholesterol (CHOL), ammonium ferrithiocyanate, sodium chloride, sodium citrate, sodium phosphate, HEPES, ethylenediaminetetraacetic acid (EDTA) and citric acid were purchased by Sigma Aldrich (USA).

### Cell lines and drug treatments

Human MM cell lines MSTO-211 H (MSTO) and NCI-H2452 (NCI) were obtained from the American Type Culture Collection (CRL-2081 and CRL-5946, ATCC, Manassas, Virginia, USA) and cultured in RPMI-1640 medium. IST-Mes2 (Mes2) was obtained from the ISTGE (HTL01007, Istituto Nazionale per la Ricerca sul Cancro – Genova, Italy) and cultured as previously described [21].

Cell lines were maintained in a humidified incubator at 37 °C in 5% CO_2_ and underwent a maximum of 15 passages after revival from frozen stocks. All cell lines were tested periodically for *Mycoplasma* contamination.

For drug treatments, cells were seeded in complete growth media 16 h before the experiments, to allow attachment but not cell-doubling and then treated with cisplatin (4.5 mg/mL) alone or combined with piroxicam (760 mM) for 24 h. In CDDP/ P combined treatment cells were pre-treated with P for 24 h before adding CDDP. In all the experiments untreated cells were used as controls.

### Mice

NU/NU CD1 male mice were purchased from Charles River Laboratories, (Calco, Italy). The animal study was performed according to the European Community Council Directive 2010/63/EU, approved by the local Ethical Committee for Animal Experiments of the ENEA, and authorized by the Italian Ministry of Health (PR 415/2021 and extension EE25E.15.EXT.0). Mice sample size has been determined using the software G*Power 3.2.1.

### Patient samples

RNAs of MM samples were collected from two different institutions in Italy and were previously described [22, 23] and underwent to a standard thoracotomy for therapeutic reasons. The patients were not treated with any previous radio or radio-chemotherapy, before to undergoing surgery. Exclusion criteria were the presence or suspicion of any infectious disease. In details, 19 unmatched samples (14 MM and 5 normal) were collected the Department of Thoracic Surgery of the Second University of Naples from 2004 to 2005. Other 17 MM samples together with the corresponding normal adjacent tissue were collected at the Oncology Clinic of the University Hospital of Ancona from 2004 to 2010. Among these samples additional 15 snap-frozen samples (normal pleura, *n* = 5, epithelioid MM, *n* = 5, and sarcomatoid MM, *n* = 5) were used as a validation set of the results obtained with the initial screening of miRNA and gene and expression.

Each subject gave a written informed consent in accordance with Italian law. Samples were processed under approval of the written consent statement by Ethical Committee of AOU of the Second University of Naples, Italy and of the University Hospital of Marche, Italy respectively.

### miRNome sequencing and data analysis

For sequencing analysis, total RNA was extracted from 1 × 10^6^ MSTO cells untreated or treated with CDDP alone or combined with P using miRNeasy mini-kit and the QIAcube (Qiagen s.r.l., Milan, Italy) following manufacturer protocol. Library preparation was done using NEBNext small RNA library prep kit (NEB, Ipswich, MA, USA) following manufacturer indications and size selection was done on a 5% non-denaturant polyacrylamide to obtain a library size of 146 nucleotides. Sequencing was performed on HiSeq2000 using a 50 nts single-end run.

For data analysis the overall content of the miRNAseq was investigated with Small RNA App (CORE App in Illumina BaseSpace cloud, (http://basespace.illumina.com/) and data were analyzed as previously described [24]. Trimmed sequences were used to calculate differentially expressed miRNAs with DESeq2 6 and the parameters chosen were FDR ≤ 0.1 and |log2FC| ≥. The statistical significance of differential expression measured by miRNA sequencing was assessed by using the R package DESeq version 3.1.1 available in Bioconductor.

Validation of selected deregulated (DEG) miRNAs was performed by TaqMan qPCR miRNA assay (Applied Biosystems) and normalized to RNU6B as indicated by manufacturer.

### In vitro analysis of miRNA 503 inhibition

Cell viability was used to analyse the effect of miR-503 inhibition on cells proliferation on MSTO, NCI and Mes2 cells. Briefly, 7.5 × 10^4^ cells/well were plated in 12-well plates and 16 h after seeding were treated with anti-miR-503 or with nc-miR-503 for 24, 48–72 h. Transfection was performed using Lipofectamine RNAiMAX, according to the manufacturer’s instructions. Subsequently, cells were collected and counted with Trypan Blue. Cell viability was also evaluated using MTT assay according to the manufacturers’ instructions. using a microplate reader (VICTOR Multilabel Plate Reader; PerkinElmer, Inc., Waltham, MA, USA).

For apoptosis detection, for each cell line analysed 1 × 10^6^ cells/well were plated in 100 mm plates and after overnight incubation, cells were treated with anti-miR-503 or with nc-miR-503 for 48 h and stained with Annexin V-FITC kit according to the manufacturer’s protocols. Apoptotic cells were determined by Flow cytometry using a FACS-Canto TM flow cytometry system (Becton Dickinson, San Jose, CA).

### Analysis of tumorigenic properties

For colony formation assay MM cells were seeded at a lower density of 1 × 10^3^ cells/well in 6-well plates and cultured for 7 days. Then, cells were treated anti-miR-503 or with nc-miR-503 for 48 h before replacing the media. Cells were grown for additional 7 days and then colonies were stained with 0.4% (w/v) crystal violet and counted. Representative plates were captured using scanner (Epson Stylus Photo, PX 650).

For wound healing assay, for each MM cell line, 3 × 10^5^ cells/well were plated in 6-well plates and after overnight incubation, gaps were created using a 200 µL pipette tip an than cells were treated anti-miR-503 or with nc-miR-503. Cell migration was monitored and photographed after 24- and 48-hours using phase contrast microscope (DMI8, Leica, Instruments, Germany). The gap closure rate was measured using Image J software (https://imagej.net/).

### LNPs formulations and in vitro analysis

Lipid nanoparticles encapsulating anti-miR-503 (LNPs-anti-miR-503), and empty LNPs were prepared as previously described [25]. The efficacy of LNPs-anti-miR-503 was confirmed in vitro by analysing the ability of loaded LNPs in reducing cell viability and inducing apoptosis, as described above. Empty LNPs were used as control.

These results and the characterization of LNPs are reported in Supplementary File (Supplementary Methods).

### In vivo analysis of the LNPs-anti-miR-503

For ectopic MM mouse model, 3 × 10^6^ MSTO cells, resuspended in 100 µl of Matrigel (BD Biosciences, CA, USA), were injected subcutis (s.c.) in the right flank of 6-week-old NU/NU CD1 male mice. Animals were housed as previously described [21]. One week after injection, mice were randomized in two experimental groups: control (empty LNPs, *n* = 7) and treated (LNPs-anti-miR-503, *n* = 7). LNPs (2 mg/Kg per mouse) were administered via intravenous (i.v.) three times a week for two weeks. Tumors masses were monitored using a caliper and the tumor volume was determined using the formula: (length × width^2^)/2. The general health status of mice and body weight was constantly monitored. At the end of experiment (day 18) mice were euthanized by cervical dislocation and tumor masses and principal organs (liver, spleen, kidney, lung, heart) were collected for further analyses. The weight of tumor masses, liver and spleen were recorded. The percentage of final tumor growth inhibition (TGI) was calculated as follows: TGI (%) = (Vc-Vt)/(Vc-Vo)*100, where Vc, Vt are the median of control and treated groups at the end of the study and Vo at the start.

### miRNA target alignment and qPCR

miRDB database (https://mirdb.org/) [26] was used to select miRNAs targets. Alignment of miRNAs predicted target was carried out using miRbase (https://www.mirbase.org) [27] and mirmap (https://mirmap.ezlab.org/) [28].

RNA extraction from MM tissues, MM cells and qPCR analysis were performed as previously described [22, 23] and run using 7900 HT Real Time PCR (Applied Biosystem). Specific primer, designed at exon–exon junctions (Primer express 2.0, Applied Biosystems) and GAPDH was used as housekeeping. Primer sequences are reported in Supplementary File (Supplementary Methods).

### Histology and immunohistochemistry

For histology, mouse tissues were stained with hematoxylin/eosin as previously described [29].

Ki67 analysis and TUNEL assay on tumor mouse tissues were performed as already described [29]. For each specimen, the score of Ki67 or the number of apoptotic cells was evaluated counting the number of positive cells visible for high power field 10 × 20. All slides were evaluated by the two blinded independent observers (A.B. and S.C.) and discordant cases were reevaluated collegially.

Immunohistochemistry (IHC) on mice tumor and human tissues specimens was performed as previously described [30, 31].

The primary antibodies used were: Rabbit anti-human Ki67 (DAKO Agilent, Santa Clara, CA USA), Rabbit anti-human BTG1 (orb35408 Biorbyt, Durham, NC, USA), Rabbit anti-human CCNG1 (orb167206 Biorbyt), Rabbit anti-human S1PR1 (EDG1) (orb350684 Biorbyt), Rabbit anti-human TIMP2 (orb543218 Biorbyt), Rabbit anti-human CXCL8 (17038-1-AP Proteintech Rosemont, IL, USA), Rabbit anti-human Osteopontin, (SPP 20416-1-AP Proteintech), Rabbit anti-human SERPINE1 (PAI-1 #49536 Cell Signaling, Danvers, MA, USA). Antibodies were used at final dilution 1:100 for 1 h at room temperature. Then, sections were incubated with UltraTek HRP secondary antibody (ScyTek Laboratories, Logan, Utah, USA) for 1 h at room temperature. Diaminobenzidine (ScyTek Laboratories, West Logan, UT, USA) was used as the final chromogen and hematoxylin was used as a contrast agent. For each tissue section, negative and positive controls were performed, either leaving out the primary antibody or using tissue expressing the antigen of interest.

Mouse tissues images were acquired by using a light microscope (Microscope Nikon ECLIPSE 55i) equipped with a Digital Image Capture software (Leica Application Suite V4.8).

Images from MM human tissues were acquired by using with an optical microscope (Zeiss, Axiocam MRc5) and staining intensity was evaluated using ImageJ.

All sections and staining were assessed by two observers (A.B. and G.G.), blinded to treatment conditions.

### Transcriptomic analysis and functional annotation

Transcriptomic analysis was performed as previously described [21] starting from 100 ng of total RNA extracted by MSTO cells treated with miR-503 inhibitor or with negative control for 48 h. Total RNA was converted in labelled ssDNA and used to hybridize Affymetrix Human Clariom S Assay (Affymetrix/Thermo Fisher), as indicated by manufacturer. Primary data analysis was performed with the TAC 4.0 (Transcriptome Analysis Console software version 3.1.0.5-Thermo Fisher) and transcriptional perturbations were detected, comparing anti-miR-503 treated cells to untreated cells.

Functional annotation of deregulated genes and Biomarker analysis were performed using Qiagen IPA software (Ingenuity Pathway Analysis, https://digitalinsights.qiagen.com).

### Statistical analysis

All the statistical analysis were performed using Graph Pad Prism 9.0 (GraphPad Software, Inc., San Diego, CA, USA). One way ANOVA was used to evaluate the significance of the differences of one variable in more than two groups. Unpaired two-tailed *t* test was used for two group comparison. Dunnett’s multiple comparison test with Bonferroni post hoc correction was used to assess the significance between each treatment group and the control group. *p* ≤ 0.05 was considered to indicate a statistically significant difference.

For in vivo experiments, tumor growth data were elaborated as temporal dynamics and statistically analyzed by regression analysis (95% CI; best fit value with 2 parameters: y-intercept and slope). Tumor mass, liver and spleen weight of each mouse were registered at the end of experiment. Data are expressed as means ± SEM for each group. *p* values were determined using two-tailed *t* test; * *p* < 0.05; ** *p* < 0.01; *** *p* < 0.001; **** *p* < 0.0001.

All the experiments were performed at least in triplicate.

## Results

### miR-503 downregulation decreases cell proliferation, migration, and invasion in mesothelioma cells

To understand if the efficacy of the CDDP/P combined treatment was carried out through miRNAs deregulation, we performed a miRNome sequencing analysis in MSTO cells after single CDDP or combined CDDP/P treatment. Differential expression analysis showed that, compared to untreated cells, both treatments deregulated about 350 miRNAs, 117 of them present in both treatments (Table S1). Among these miRNAs we focused our attention on those with stronger deregulation in the combined treatment. The resulting 22 miRNAs (Table S1) were analysed bioinformatically to select the miRNAs whose predicted target genes were connected directly or indirectly to p21, since the anticancer effect of CDDP/P treatment was associated to nuclear localization of p21 [32].

Target prediction using miRDB database allowed us to select three miRNAs, miR-7-1 miR-139 and miR-503, showing high scores for predicted target genes related to cell cycle. Specifically, miR-7-1-3p was predicted to target CCNG2, CDKN2B and BCCIP (target scores 100, 96, 92 respectively); miR-139-5p was predicted to target CDC42 and PAK2 (target scores 68 and 67) and miR-503-5p was predicted to target CCND2, CCND1, CDKN2AIP (target scores 98, 96, 81 respectively).

Data from sequencing analysis showed that after CDDP/P treatment, miR-7-1 and miR-503 resulted downregulated, while miR-139 resulted upregulated.

To validate their expression q-PCR analysis was performed in MSTO and NCI cells, two cell lines sensitive to the combined treatment, and in Mes2, a cell line resistant to it for the absence of p21 [32].

**Fig. 1:**
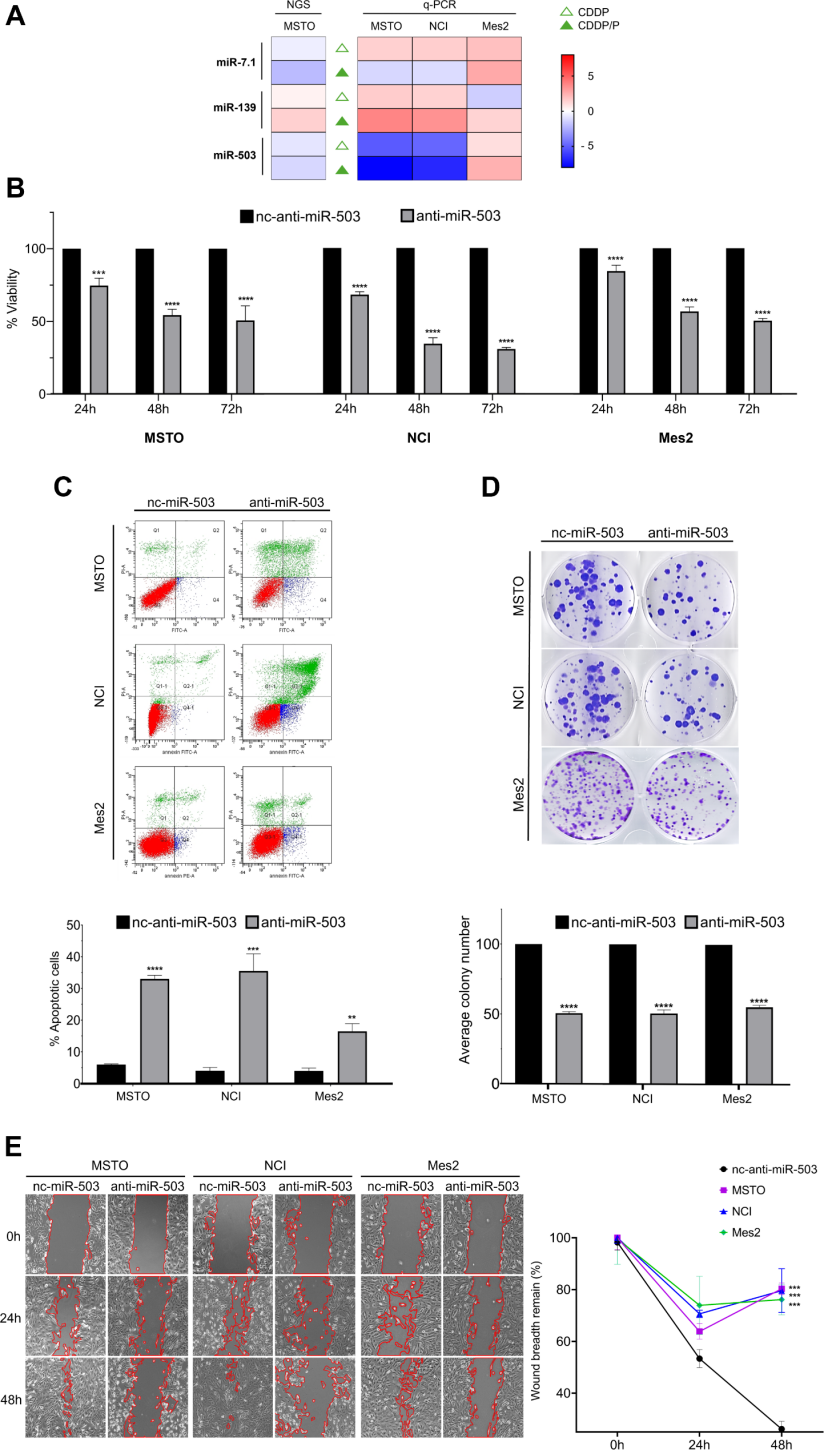
miR-503 downregulation affects cancer properties in MM in vitro. **A** NGS expression values of DEG miRNAs selected after single CDDP and combined CDDP/P treatments and q-PCR validation in MM cells. **B** Effects on cell viability of miR-503 inhibition at different time. 48 h treatment resulted the best time and reduction was 46% MSTO, 66% NCI and 43% Mes2. A lower decrease was detected after 24 h (75% MSTO, 68% NCI and 84% Mes2) while after 72 h the effect was comparable to the 48 h (49% MSTO, 69% NCI and 50% Mes2). **C** FACS analysis confirms that cell viability decrease after 48 h treatments is due to apoptotic increase in all MM cells treated with anti-miR-503 (33% MSTO, 35% NCI and 16% Mes2) compared to nc-anti-miR-503 (6% MSTO, 4% NCI and 4% Mes2), used as control. Histograms report a data summary of the apoptotic index for both treatments. **D** Crystal violet colony assay in MM cells transfected for 48 h with anti-miR-503 or with negative control (nc-anti-miR-503). Cells were grown for 7 days before transfection and after additional 7 days the colonies were stained with 0.4% (w/v) crystal violet and counted using Image J. Reduction of colony numbers were: 50% MSTO, 50% NCI and 45% Mes2. Data are shown in the histogram. **E** Wound-healing assay shows that miR-503 inhibition after 48 h greatly reduced the migration capability of MM cells. The graph reports the residual gap in treated anti-miR-503 cells compared to control (80% MSTO, 80% NCI and 76% Mes2). Data are presented as the mean *±* SD of at least three independent experiments (*n* = 3). **p < 0.01 ***p < 0.001 ****p < 0.0001 vs. control

Results showed that only miR-503 expression was linked to the treatments (Fig. 1A) and that in Mes2 miR-503 showed an opposite expression. This result suggested that miR-503 could play a role in the apoptosis induction associated to the combined treatment.

To explore the bioactivity of miR-503 inhibition in mesothelioma, we tested in vitro if its downregulation could induce apoptosis and affect cancer properties. To this aim we performed a series of experiments in MM cell lines.

First, we analysed the effect of miR-503 inhibition on cell viability in a time course analysis. Results clearly indicated that 48 h was the best time-treatment determining a decrease in cell proliferation of about 50%. No effect was observed after nc-miR-503 transfection (Fig. 1B).

To deepen understand if this reduction was due to increased apoptosis, we performed a cytofluorimetric analysis after 48 h of treatments. FACS analysis showed an increase of apoptotic cells of about 30% in all cell lines tested (Fig. 1C).

Then we analyzed the effects of miR-503 inhibition on self-renewal capacity and cell migration ability. As shown in Fig. 1D, transfection of anti-miR-503 determined a marked reduction of colony number formation in MM cells, thus suggesting that its expression can be associated to cancer growth. Similarly, wound-healing assay showed that inhibition of miR-503 significantly affected the migration capability of mesothelioma cells. In fact, in anti-miR-503 treated cells the wound gap closure was about 80% after 48 h, while it appeared complete in cells treated with nc-miR-503 (Fig. 1E).

### miR-503 downregulation decreases tumor growth in vivo

To investigate in vivo the anticancer effect of miR-503 inhibition, we took advantage of using LNPs as an efficient and safe delivery system that was previously described [25]. Empty and loaded with anti-miR-503 LNPs showed a similar diameter and negative superficial charge indicating that the encapsulation of miR-503 inhibitor did not influence vesicles size. To ascertain that LNPs preserved the biological activity of anti-miR-503, we performed preliminary in vitro assays on MSTO cells. Analysis of cell viability confirmed that LNPs-anti-miR-503 resulted more effective after 48 h of treatment, when cell decrease was reduced of about 50%. In addition, FACS analysis confirmed that the decrease was due to apoptosis. No effect was evidenced after treatment with empty LNPs (Fig. S1). These results confirmed the efficacy of the delivery system and allowed us to set up the experiments to evaluate in vivo the anticancer effect of miRNA-503 inhibition using a murine ectopic model of mesothelioma.

**Fig. 2:**
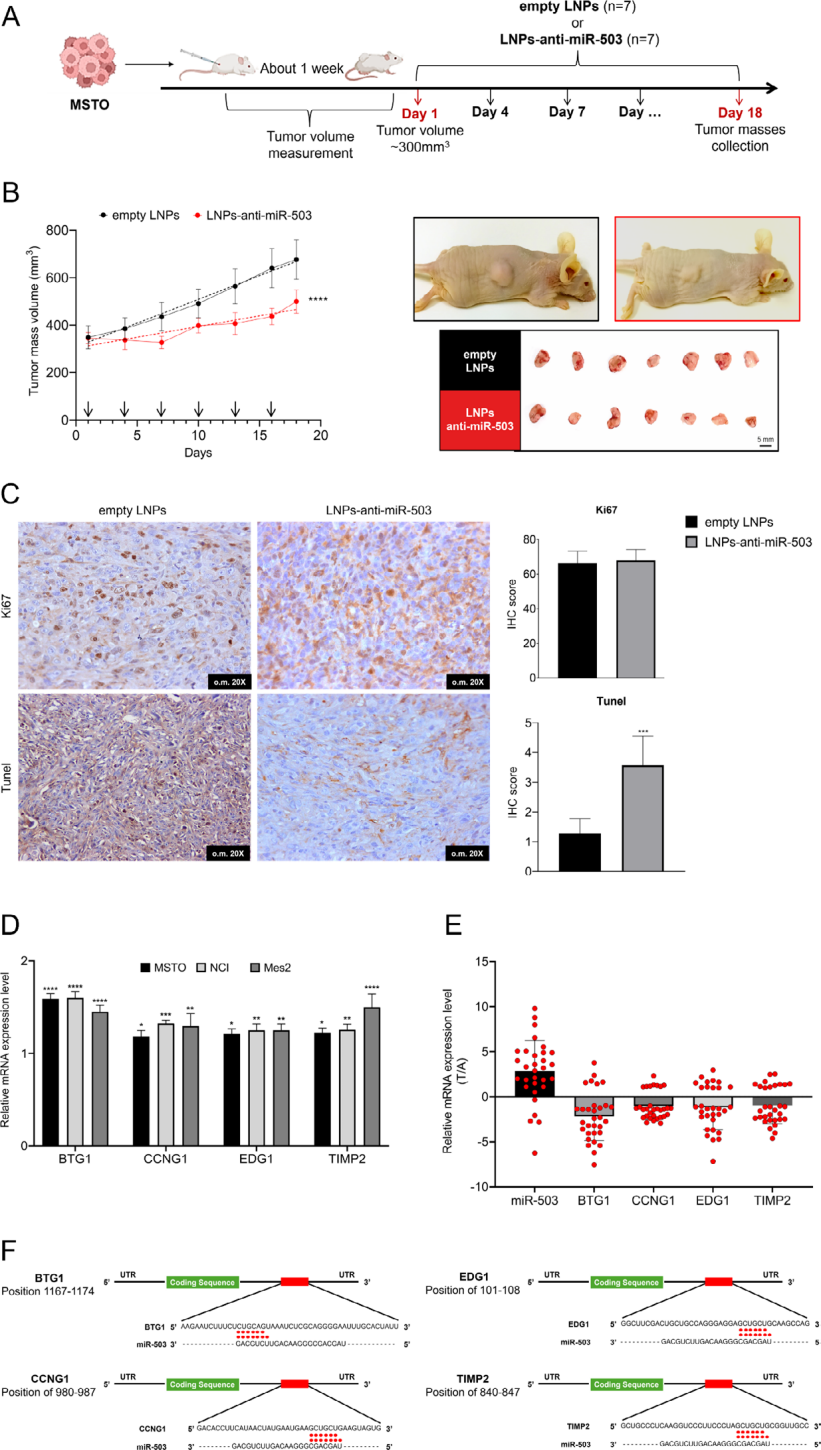
In vivo anticancer effects of miR-503 inhibition. **A** Experimental scheme showing design and details of the mesothelioma xenograft mouse model of MM. Treatments was performed inoculating i.v., after tumor growth, LNPs-anti-miR-503 or empty LNPs. **B** Tumor growth reduction evaluated by analysing tumor masses volume at the days of treatment. Data are expressed as tumor mass volume means ± SEM for each group and the linear regression (dashed line) is reported. Images of tumor masses from mice treated with LNPs-anti-miR-503 or with empty LNPs at the end of experiment are shown. **C** Effects of miR-503 inhibition on cell proliferation (Ki67) and apoptosis (TUNEL) in tumor masses compared to control (empty LNPs) (original magnification 20X). Bar plots indicates no changes for Ki67 and higher apoptotic index in tumors treated with LNPs-anti-miR-503 compared to empty LNPs, confirming the pro-apoptotic role of miR-503 inhibition in vivo. **D** Expression analysis of miR-503 putative target genes by q-PCR in MM cells after miR-503 inhibition **E** q-PCR analysis in human MM samples of the miR-503 putative target genes confirms their inverse expression and the concomitant up-regulation of miR-503. **F** Base pair alignment among miR-503 and the 3’UTR region of the putative target genes. Data are presented as the mean *±* SD of at least three independent experiments (*n* = 3) for the in vitro experiments while for the in vivo analysis numbers of samples are reported in Methods. *p < 0.05, **p < 0.01 ***p < 0.001 ****p < 0.0001 vs. control

Male nude CD1 mice were s.c. inoculated with MSTO cells, and one week after implantation, when tumor masses reached about 350 mm^3^, mice were randomized in two groups and treated intravenously with of empty LNPs or with LNPs-anti-miR-503 (2 mg/kg) every three days for a total of 6 injections as described in the experimental scheme (Fig. 2A). Results showed that empty LNPs did not exert any anticancer effect, while LNPs-anti-miR-503 determined progressive reduction of tumor growth with a reduction of 12% already evidenced after the first administration that reached 53.4% at the end of the experiment (Fig. 2B). The anticancer activity of miR-503 inhibition in vivo was also supported by the reduction of tumor weight in treated mice (Fig. S2A). Importantly, no significant variation was observed in mice during the experiment in total body weight (Fig. S2B) or in liver and spleen weight (Fig. S2C, D), suggesting a good tolerance of LNPs anti-miR-503 administration supported also by histopathological analysis of principal organs collected (data not shown).

Histopathological analysis of ectopic tumors showed that treatment with LNPs-anti-miR-503 caused partial substitution of the tumor tissue by calcified and necrotic tissue (Fig. S3). Tumor tissue from anti-miR-503 treated mice displayed higher apoptotic index, as evaluated by TUNEL assay compared to samples from mice treated with empty LNPs (Fig. 2C). The analysis of the proliferation index, evaluated by Ki67 nuclear expression, showed no significant differences between the two groups reinforcing the pro-apoptotic role of miR-503 inhibition in MM.

### miR-503 expression analysis in mesothelioma tissue samples and target genes

To identify the putative miR-503 target genes involved in MM tumor growth we analysed the biomarker gene list previously reported in MM tissues by our group [22]. Among them, we selected seven genes associated to cell proliferation and apoptosis that resulted downregulated in tumor samples: BTG1, CCNG1, DMD, EDG1, SEMA3G, SYNPO2, TIMP2.

Their expression was analysed by q-PCR analysis in MM cells after miR-503 inhibition. Results confirmed the expression only for BTG1, CCNG1, EDG1, TIMP2. The same analysis performed in human MM samples confirmed in vivo an opposite expression of miR-503 and of the putative target genes with upregulation of miR-503 and a concomitant decreased expression of the selected genes (Fig. 2D, E). These data reinforced the possible biological role played by this miRNA in mesothelioma and confirmed that expression on miR-503 is linked to decreased expression of genes involved in apoptosis.

The specific miRNA tools miRbase and mirmap were then used to analyze the base-pairing among miR-503 and the 3’UTR region of these genes. Results supported the q-PCR data confirming the presence of the base pairing and the target site accessibility, the most predictive feature to identify a candidate gene target (Fig. 2F).

**Fig. 3:**
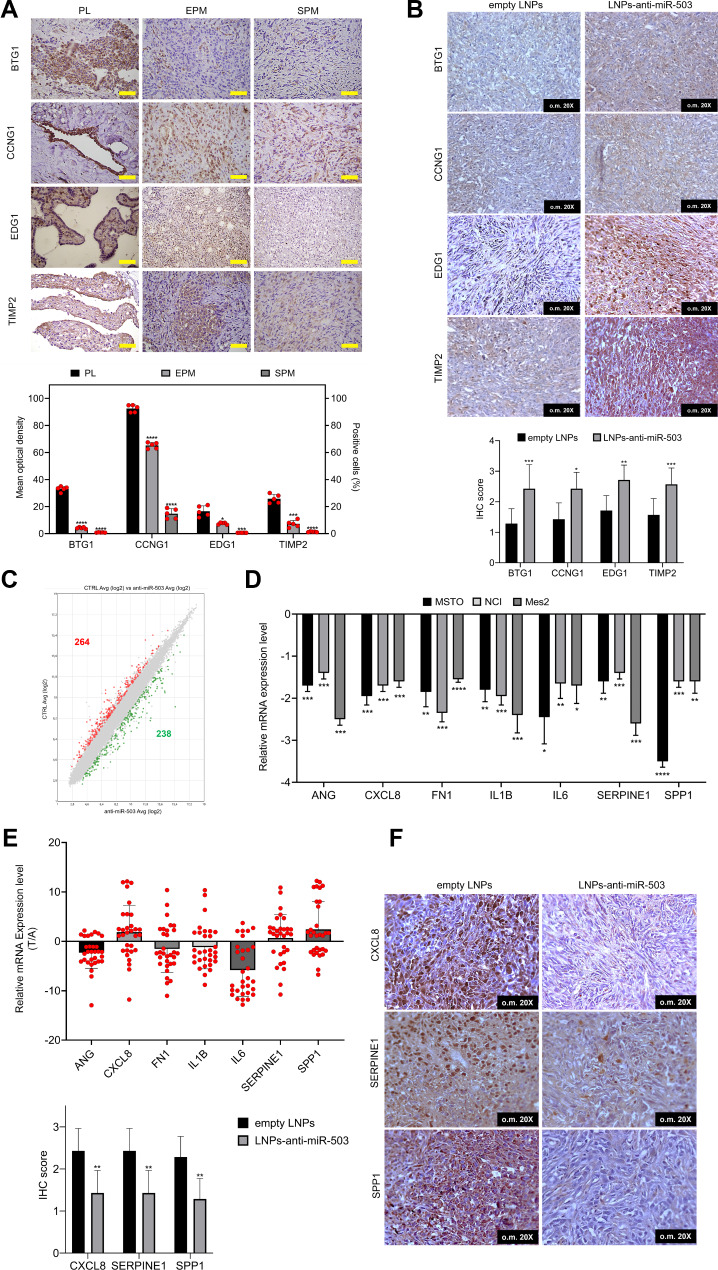
Histological analysis of miR-503 target genes and transcriptomic analysis of miR-503 inhibition. **A** Evaluation of the expression of BTG1, CCNG1, EDG1 and TIMP2, in normal pleural (PL) and pleural mesothelioma tissues (EPM epithelial istotype; SPM, sacomatoid istotype). Yellow Scale bar = 100 μm. Data shown are mean ± S.D. of five independent experiments. **B** IHC analysis of the putative miR-503 target genes on mice tumors treated with LNPs-anti-miR-503 or empty LNPs (original magnification 20X). **A** and **B** confirm the in vitro results. **C** Transcriptome analysis in MSTO cells after miR-503 inhibition detected about 500 deregulated genes (red upregulated, green downregulated). **D** Expression analysis of MM biomarkers deregulated by inhibition of miR-503 in MM cells. **E** q-PCR analysis in human MM samples of the miR-503 MM biomarkers confirms the inverse expression only for CXCL8, SERPINE1 and SPP. **F** IHC analysis of the miR-503 deregulated MM biomarkers on mice tumors treated with LNPs-anti-miR-503 or empty LNPs (original magnification 20X). For each IHC panel bar plots report the score for the samples analysed. Data presented as the mean *±* SD of at least three independent experiments (*n* = 3) for the in vitro experiments while for the in vivo analysis numbers of samples are reported in Methods. *p < 0.05, **p < 0.01 ***p < 0.001 ****p < 0.0001 vs. control

In addition, protein expression analysis of BTG1, CCNG1, EDG1, and TIMP2 in MM tissues in mouse tumor masses treated with LNPs-anti-miR-503 confirmed the q-PCR results and the opposite expression of miR-503 and of its target genes also in vivo (Fig. 3A, B).

### Transcriptome analysis shows that inhibition of miR-503 affect the expression of mesothelioma biomarkers

To investigate if miRNA-503 inhibition could modulate known biomarkers specifically linked to mesothelioma, we performed a transcriptome analysis by means of Clariom arrays in MSTO cells treated with miR-503 inhibitor for 48 h. The analysis detected a total of 264 upregulated and 238 downregulated genes (Fig. 3C and Table S2). Deregulated genes were functionally annotated through IPA analysis with the aim to highlight the major molecular pathways associated to the miR-503 inhibition. Notably, the analysis showed that among the “Molecular and Cellular Functions” most of deregulated genes were associated to cell death and proliferation (Fig. S4). Then to better analyze the putative role of miR-503 in the context of MM we performed a “Biomarker analysis” with the aim of identifying mesothelioma biomarkers associated to miR-503 inhibition. This analysis based on mechanistic connection to diseases and the detection in body fluids, allows to identify and prioritize the most relevant molecular biomarker candidates discriminating a disease state and/or drug response.

The analysis retrieved 42 putative biomarkers all downregulated and associated to cancer (Table S2). Among them we selected those genes reported associated to MM with an extracellular location, a feature that allows an easy detection and that could be informative for personalized treatment: ANG, CXCL8, FN1, IL1, IL6, SERPINE1 and Osteopontin (SPP1). Their expression was validated by q-PCR in MM cell lines after miR-503 inhibition and the results confirmed their downregulation (Fig. 3D). However, in vivo analysis on human mesothelioma samples confirmed a reverse expression of CXCL8, SERPINE1, and Osteopontin, while the expression values were not confirmed for the remaining genes (Fig. 3E). Interestingly, the putative biomarkers whose expression was confirmed in both cells and in human samples are known to be associated to MM. According to these results, IHC analysis of mouse tumor tissues, confirmed that miR-503 inhibition was able to significantly reduce their expression (Fig. 3F).

## Discussion

Asbestos exposure is known to be the main carcinogen involved in mesothelioma development. Despite the poor efficacy of drug treatments in MM, the use of platinum compounds combined with other drugs is considered the first-line therapy. However, the development of drug resistance remains the major challenge in chemotherapy.

In the latest years different miRNAs have been considered as novel cancer drug targets also in MM [33]. These miRNAs, by acting as oncogenic or as tumor suppressor are able to inhibit tumor growth [34]. Recently, different therapeutic strategies are based on the use of miRNAs as novel tools to treat cancer, and different miRNAs have been tested in phase I and II clinical trials [4]. In addition, miRNAs are currently considered more attractive tumor targets since their deregulation has been associated to platinum drug sensitivity and to the development of tumor chemoresistance also in MM [35].

In the present study we investigated if the efficacy of CDDP/P combined treatment in MM was related to specific miRNA deregulation. We identified miR-503 that acts as an oncomiR, as the molecular player involved in the cancer growth inhibition triggered by this treatment.

miR-503, is a member of the miR-16 family, which includes other 5 members (miR-15a/b, miR-16, miR-195, miR-424, and miR-497) [36] that can act as oncogene or tumor suppressor depending on the cellular context [37, 38]. For example, in MM miR-16 acts as tumor suppressor [10] while miR-503 displays different functions depending on cancer or cell type. It is upregulated in different human cancers while in others, such as colon cancer, it has an oncogenic role and confers platinum resistance [39].

Our results showed that inhibition of miR-503 expression in vitro induced apoptosis and reduced cancer properties in two different mesothelioma cell lines and that similar effects were detected also in Mes2 cells that does not respond to the CDDP/P combined treatment.

The anticancer efficacy of miR-503 inhibition was evaluated in vivo in an ectopic mouse model of MM using LNPs to encapsulate and deliver anti-miR-503. LNPs were chosen as they are the most effective and safe delivery system being also used in humans for drugs and vaccine delivery [40].

In vivo results confirmed the oncogenic role of miR-503 in MM since in mice intravenous injection of LNPs containing anti-miR-503 significantly reduced tumor growth and determined apoptotic increase. Interestingly, the reduction in tumor growth in vivo was independent from the proliferation activity of tumor cells, since Ki67 score was not changed in treated animals respect to the control. Indeed, it was completely dependent on apoptosis, thus confirming the in vitro data.

Further investigation of miR-503 expression in MM tumor samples showed that it was upregulated in the tumor tissues compared to the normal ones, suggesting that it could be involved also in vivo in MM progression.

Beyond the oncogenic role of miR-503 in MM, we wondered if this miRNA could target genes linked to cell proliferation. To this aim we leveraged the biomarker genes deregulated in MM previously reported by our group [22], focusing our attention to downregulated genes associated to cell proliferation and apoptosis. Among them, seven putative genes were selected: BTG1, CCNG1, DMD, EDG1, SEMA3G, SYNPO2, TIMP2. However, q-PCR analysis after miR-503 inhibition in MM cells confirmed the inverse expression only for BTG1, CCNG1 EDG1 and TIMP2. According to this, we found that the 3’UTR regions of these genes showed a match with the miR-503 seed sequence.

In addition, expression analysis of these genes in human MM samples confirmed their inverse regulation compared to miR-503. Their expression was further confirmed at protein level in both ectopic treated tumor samples and in human MM tissues. These results reinforce the oncogenic role of this miRNA in mesothelioma.

The expression of the putative miR-503 target genes strongly correlates with tumor growth, and, except for TIMP2, they have never been associated with MM.

BTG1 is a tumor suppressor gene that inhibits cell proliferation and whose decreased expression is associated with malignant progression and poor prognosis [41].

CCNG1 and EDG1 both show a dual role in cancer since they can positively or negatively regulate cell growth. The cyclin gene CCNG1 has been reported downregulated and related to a worse overall survival in several cancers [42].

The G protein-coupled receptors encoded by EDG1 is involved in several processes such as cell survival, migration, and angiogenesis. EDG1 decreased expression has been linked to poor prognosis in breast and lung cancer [43].

Decreased expression of TIMP2 in MM has been associated with tumor progression and low survival [44]. TIMP2 encodes endogenous inhibitor of the matrix metalloproteinases that plays a key role in the maintenance of tissue homeostasis. An imbalance between matrix metalloproteases and their endogenous inhibitors leads to degradation of extracellular matrix and promotes tumor invasion and metastasis [45].

The identification of putative miR-503 target genes never described in MM prompted us to investigate if miR-503 deregulation could be linked to the expression of specific MM biomarkers. To this aim, we analysed the transcriptomic perturbations in MSTO cells after its inhibition. Functional analysis confirmed the role of miR-503 since deregulated genes were mainly associated to cell death and proliferation. Subsequent biomarker analysis retrieved 42 putative biomarkers, among which we selected downregulated genes with an extracellular location and previously associated to MM: ANG, CXCL8, FN1, IL1, IL6, SERPINE1 and Osteopontin. Their expression was confirmed in MM cell lines after miR-503 inhibition, while in human mesothelioma tissues we found a reverse expression only for CXCL8, Serpine1, and SPP1 compared to the cells. IHC on mouse treated tumor samples confirmed their downregulation. These results are very striking since all these biomarkers are known to be associated to MM.

In this study we described a novel role of miR-503 in MM that acts as possible mediator of tumor growth both in vitro and in vivo, and reported for the first time an oncogenic role for a member of miR-16 family in MM.

## Conclusions

miRNAs regulate a wide range of cellular processes such as stem cell maintenance, hematopoiesis, proliferation, and differentiation but they also are involved in tumorigenesis. In cancer, miRNAs can play a dual role acting as oncomiR or tumor suppressor, through regulation of the expression of genes encoding tumor suppressor proteins [5, 7].

Our study suggests as an important oncogenic role for miR-503 which is closely involved in tumor growth and metastasis in MM.

Our results indicate that cisplatin combined to piroxicam in MM can act through specific miRNAs modulation. The relevance of our study is remarkable due to the expected increase of MM diagnosis in the near future and to date no cures are available.

The novelty of our study consists in combining the use of a therapeutic miRNA in MM with a safe delivery system, two aspects that have reached high levels of safety, being routinely approved for different applications. In the future, understanding how miR-503 inhibition achieves its anticancer effects could reveal new therapeutic strategies for MM treatment.

## Electronic supplementary material

Below is the link to the electronic supplementary material.

Supplementary Material 1

Supplementary Material 2

Supplementary Material 3

## Data Availability

Transcriptomics data generated were deposited in Gene Expression Omnibus (GEO) repository with the accession number GSE269532.

## Abbreviations

CDDP: Cisplatin
LNP: Lipid Nano Particles
MM: Malignant mesothelioma
P: Piroxicam
