# Supplementary material for: miRNA-503 inhibition exerts anticancer effects and reduces tumor growth in mesothelioma
Source: J Exp Clin Cancer Res. 2025 Feb 22;44:65. doi: 10.1186/s13046-025-03283-0 (PMC11846362; doi:10.1186/s13046-025-03283-0)
Supplement: Supplementary file 1 — Supplementary Material 1 [file 13046_2025_3283_MOESM1_ESM.docx]

**Supplementary File**

**Methods**

1. **LNPs characterization**

The mean size, population distribution (PI) and superficial charge (ζ) of LNPs-anti-miR-503 were performed by dynamic light scattering after samples dilution (1:100 v/v) by Nanosizer Ultra (Malvern, UK). Results analyses were calculated as the mean of measures carried out on at least three different batches (n=3).

The amount of anti-miR-503 encapsulated into the LNPs was determined spectrophotometrically. Briefly, an aliquot of the LNPs formulation was dissolved in methanol (1:100 v/v) and samples were centrifugated for 30 min at 13000 rpm (MIKRO 20; Hettich, Tuttlingen, Germany). The supernatants were analyzed by UV (UV-1800, UV Spectrophotometer) at the wavelengths of 260 nm. The amount of anti-miRNA loaded into the nanocarriers was expressed as anti-miRNA encapsulation efficiency (EE %), calculated as % ratio between anti-miRNA actual loading and anti-miRNA theorical loading in formulation (nmol of anti-miRNA/mg of total lipids). For each formulation, the results were calculated as the mean of the measures obtained in three different batches (n=3).

As shown below, empty LNPs, prepared without anti-miR-503 had a mean diameter of about 106.5 nm and a negative superficial charge of -27.1 mV. The encapsulation of anti-miR-503 did not influence vesicles size that results of about 102 nm. All the formulations were characterized by a narrow size distribution with a PI < 0.2 and a negative zeta potential (ZP) (about -30 mV).  Finally, the actual loading of anti-miRNA-503 was 25.4 nmol for mg of total lipids, corresponding to an anti-miRNA encapsulation efficiency of about 92 %.

| **Formulation** | **Diameter**  **(nm) ± SD** | **PI ± SD** | **ZP**  **(mV ± SD)** | **anti-miR-503 theoretical loading (nmol/mg lipids)** | **anti-miR-503**  **actual loading (nmol/mg lipids) ± SD** | **EE**  **(% ±  SD)** |
| --- | --- | --- | --- | --- | --- | --- |
| **LNP** | 106.5 ± 1.6 | 0.1 ± 0.01 | -27.1 ± 2.0 | - | - | - |
| **LNP  1** | 102.0 ± 4.3 | 0.1 ± 0.01 | -30.2 ± 0.4 | 27.5 | 25.4 ± 0.3 | 92.3 ± 1.0 |

1. **Gene primer sequences used for q-PCR analysis:**

| **Gene** | **Forward** | **Reverse** |
| --- | --- | --- |
| ANG | CCC GTT TCT GCG GAC TTG T | GCC CAT CAC CAT CTC TTC CA |
| BTG1 | GCT GGC AGA ACA TTA TAA ACA TCA CT | GGT AAC CCG ATC CCT TGC A |
| CCNG1 | TGC AAC TGT ATT ATT CAC TCC TTC AAG | CCT TCA GTT GAG CTT CTA GTC TTT CA |
| CXCL8 | TGT CTG GAC CCC AAG GAA AA | CTC AGC CCT CTT CAA AAA CTT CTC |
| EDG1 | GAG CGA GGC TGC GGT TT | GGT GGT TCG AGT GAT CCA |
| FN1 | CGA GGA GAG TGG AAG TGT GAG A | GGT AAA CAG CTG CAC GAA CAT C |
| GAPDH | CAA GGC TGT GGG CAA GGT | GGA AGG CCA TGC CAG TGA |
| IL1B | CAG GCT GCT CTG GGA TTC TC | CAT GGC TGC TTC AGA CAC TTG |
| IL6 | CCT GCA GAA AAA GGC AAA GAA | CAG GCT GGC ATT TGT GGT T |
| Serpine 1 | AGC CAC TGG AAA GGC AAC AT | GGA GAA CTT GGG CAG AAC CA |
| SPP1 | GTT TCG CAG ACC TGA CAT CC | TCC TCG CTT TCC ATG TGT GA |
| TACC1 | GCA AGC CGA GAC CAA ATC C | AGC TTC AGG AGT CTC AAA ATT ACC TT |
| TIMP2 | CCA AGC AGG AGT TTC TCG AC | GAC CCA TGG GAT GAG TGT TT |

**Results**

**
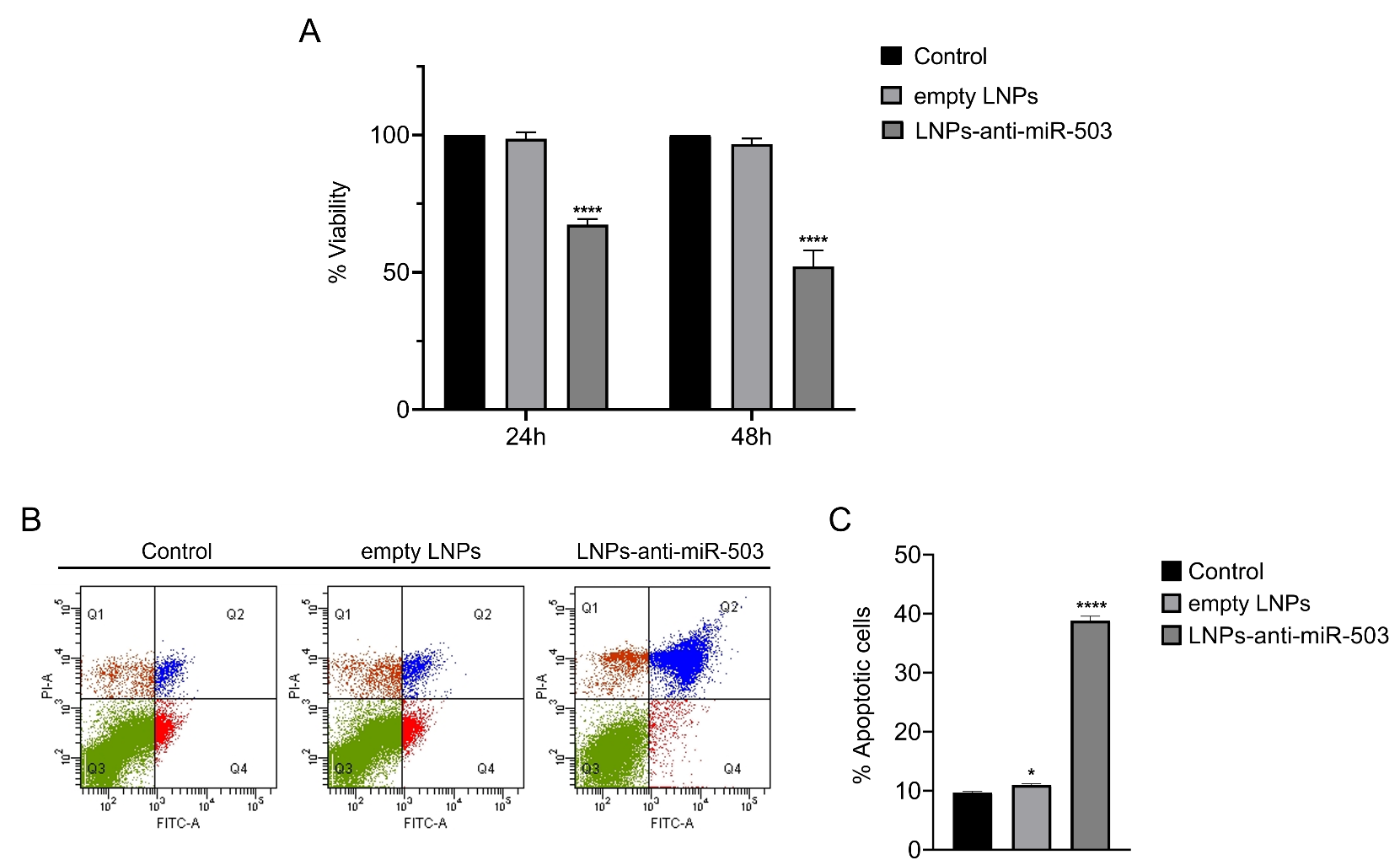
**

**Figure S1.**

**LNPs preserved the biological activity of anti-miR-503. A** MSTO cells were treated with LNPs empty or loaded with anti-miR-503 for 24 and 48 hours. 24 hours of treatment with LNPs-anti-miR-503 determined a reduction in cell viability of 33% while 48 hours determined a reduction of 48%, confirming that 48 hours was the best treatment time also fot LNPs miRNA delivery. The safety of the empty LNPs was confirmed by the absence of cytotoxicity (1% and 5% at 24 and 48 hours of treatment respectively). **B** FACS analysis confirmed the apoptosis rate in MSTO cells treated with loaded LNPs for 48 hours compared to empty LNPs. **C** Histograms report a data summary of the apoptotic index of LNPs-anti-miR-503 treated cells (11% and 39% in cells treated with empty or loaded LNPs respectively) compared to untreated cells (Control). Data are presented as the mean *±* SD of at least three independent experiments (n = 3). * p = 0.01, **** p < 0.0001 vs. control.

**
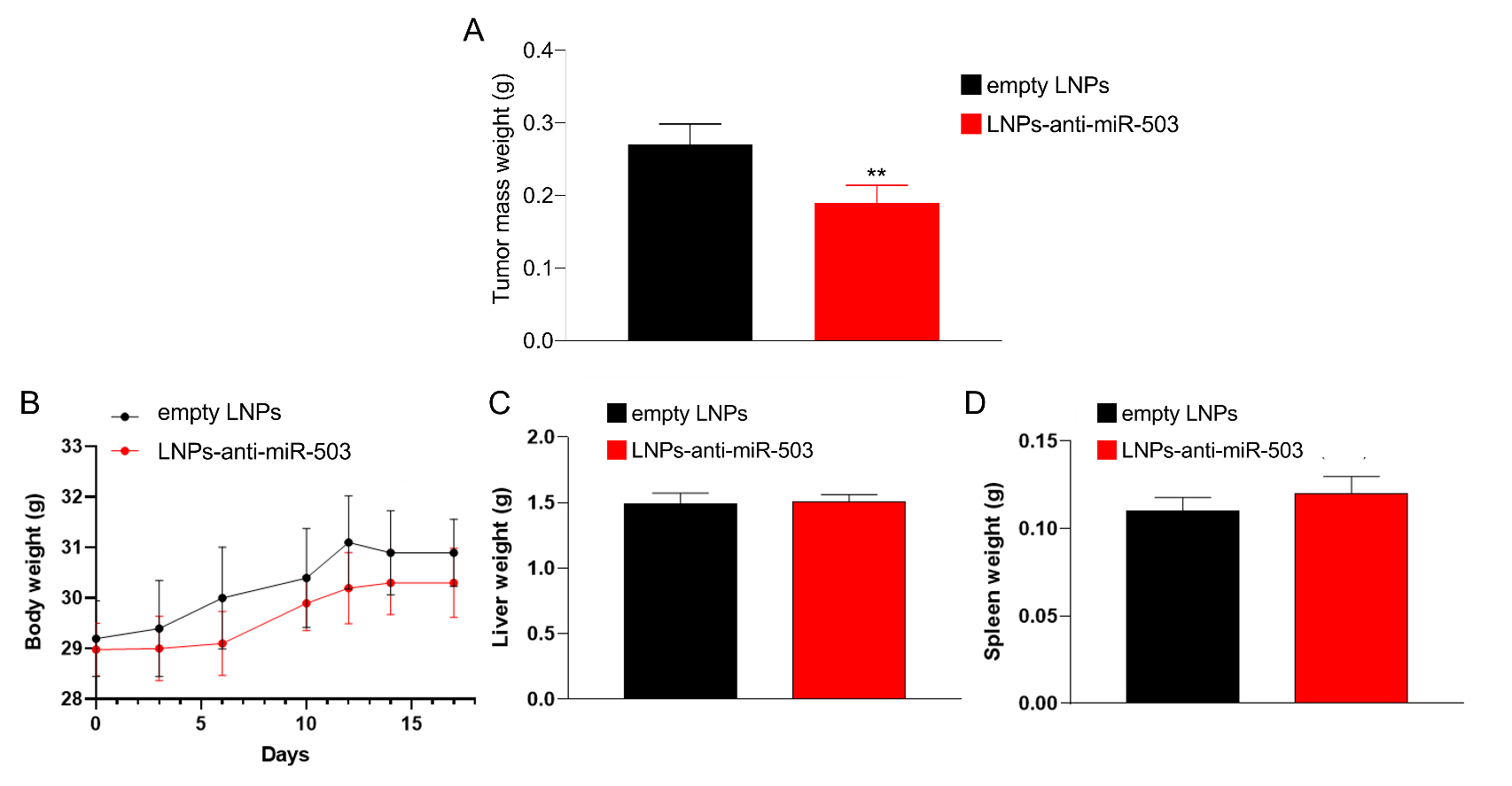
**

**Figure S2.**

***In vivo* anti-miR-503 anticancer activities.**

**A** Histogram reporting the weight of the tumor masses from xenograft mouse model of MM. **B** mice body weight of both experimental groups recorded from the beginning of the experiment at the day of treatments. **C** and **D** Livers and spleens weight indicate the good tolerance of the delivery system. Data show the means ± SEM of livers and spleens weight respectively for each experimental group.

* p< 0.05; ** p< 0.01; *** p< 0.001; **** p< 0.0001.

empty LNPs


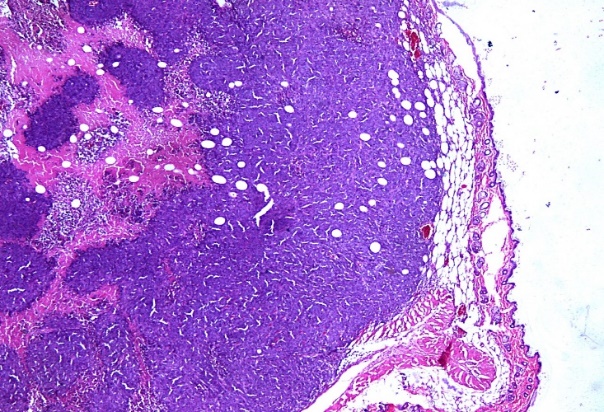

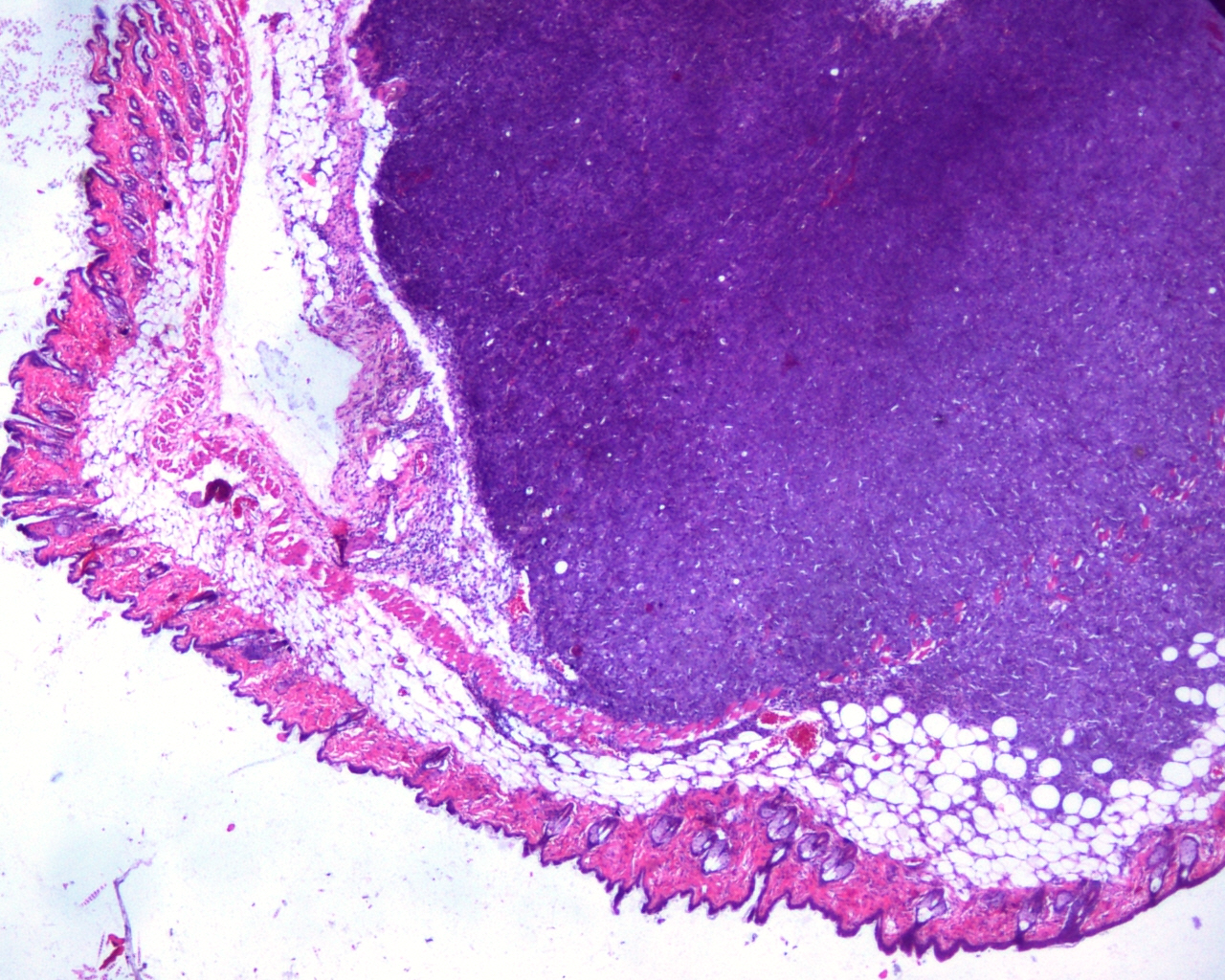


LNPs-anti-miR-503

**Figure S3.**

Histopathological analysis of necrosis in ectopic tumors. Hematoxylin/eosin staining of mice tumor tissues indicate that LNPs-anti-miR-503 caused partial substitution of the tumor tissue by calcified and necrotic tissue (original magnification 20X). Representative image of tumor treated with empty or LNPs-anti-miR-503 is shown.


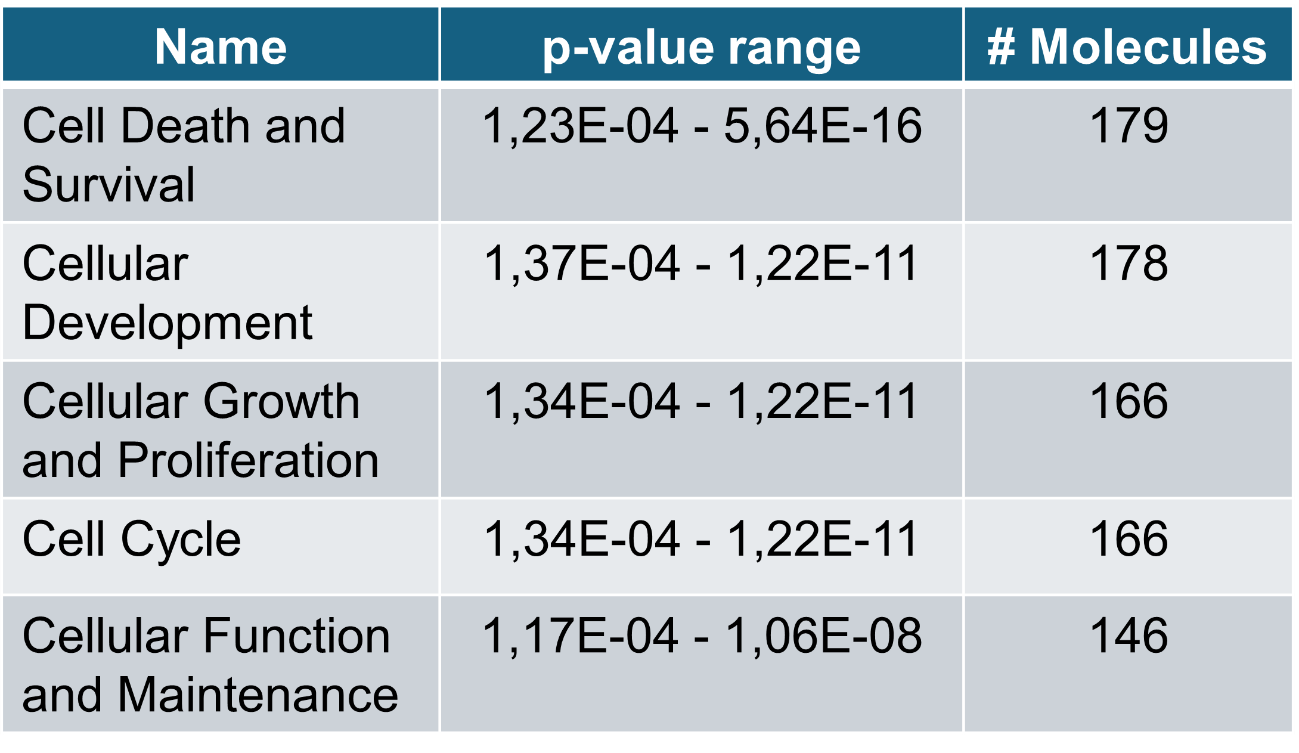


**Figure S4.**

IPA top “Molecular and Cellular Functions”. Functional annotation of DEG genes after miR-503 inhibition indicates that most of them are associated to cell death and proliferation.
